# Epidemiological and Clinical Characteristics of the Course of COVID-19 Among Vaccinated and Unvaccinated Heart Transplant Recipients in Slovenia

**DOI:** 10.3390/vaccines12121366

**Published:** 2024-12-03

**Authors:** Nina Grasselli Kmet, Matej Mavrič, Rajko Saletinger

**Affiliations:** 1 Infectious Diseases Department, University Medical Centre Ljubljana, 1000 Ljubljana, Slovenia; matej.mavric@kclj.si (M.M.); rajko.saletinger@kclj.si (R.S.); 2 Faculty of Medicine, University of Ljubljana, 1000 Ljubljana, Slovenia

**Keywords:** heart transplant recipients, COVID-19, clinical course, vaccination, hesitancy

## Abstract

**Background**: Patients receiving heart transplantation require lifelong immunosuppression and compared to the general population, they have a more than five times higher chance of acquiring COVID-19, and their mortality rates are higher. The aim of the present study was to estimate the epidemiological and clinical characteristics of COVID-19 in heart transplant recipients (HTRs) in Slovenia to estimate the vaccination rate and evaluate possible vaccination-hesitant subgroups. **Methods**: All SARS-CoV-2-positive HTRs (N = 79) between 1 March 2020 and 31 December 2023 at the Infectious Diseases Department, University Medical Centre Ljubljana, Slovenia, were included retrospectively. Demographic, clinical and vaccination data were extracted from medical documentation and a statistical evaluation was performed. **Results:** The observed vaccination rate was 63.3%, but among patients who received transplants before the pandemic, it was statistically significantly higher (*p* = 0.027). Vaccinated HTRs were statistically significantly older (*p* = 0.004) and had a significantly higher Charlson Comorbidity Index (*p* = 0.018). Our results indicate no significant differences between vaccinated and unvaccinated HTRs regarding acute respiratory insufficiency (*p* = 0.135), length of hospital stay (*p* = 0.106), intensive care unit admission (0.414) and in-hospital mortality (*p* = 0.317), but we observed statistically more frequently an asymptomatic course in those vaccinated (*p* = 0.050), and a longer length of stay in vector vaccine recipients (*p* = 0.011) and in those not re-vaccinated (*p* = 0.030). There was a significantly higher re-vaccination rate in males (*p* = 0.005). **Conclusions**: An asymptomatic course of COVID-19 was more often observed in vaccinated HTRs. Our findings suggest statistically significant differences in COVID-19 vaccine acceptance rates; younger HTRs and those transplanted after the pandemic are more hesitant to vaccinate, while females accept booster doses less frequently.

## 1. Introduction

Patients receiving solid organ transplantation (SOT) require lifelong immunosuppression, making them more susceptible to COVID-19, as multiple immunosuppressive drugs inhibit cellular and antibody-mediated immunity. In addition to immunodeficiency, HTRs often have comorbidities, which further affect the severity of the disease. There are few data on the prevalence and clinical features of COVID-19 in HTRs. The data suggest that compared to the general population there is a more than five times higher chance of acquiring COVID-19 in HTRs, the chances of hospitalization are approximately 80% and the mortality rates range from 10% to 30% [1,2,3,4]. In 2022, there were 2444 heart transplants in Europe and, according to Slovenian Eurotransplant data, there were 21 heart transplants in Slovenia [5]. Since 2018, all heart transplants in Slovenia have been performed at UMC Ljubljana. Before 2018, Slovenian patients mostly received transplants in Vienna, Austria [6]. While vaccination against COVID-19 is recommended for HTRs [7,8], the effectiveness of the vaccine might be reduced due to chronic immunosuppressive therapy [9,10,11,12]. Despite the relatively poor response to the vaccine, data from studies comparing the clinical course of COVID-19 in vaccinated and unvaccinated HTRs are sparse, but they show a significantly more favorable course in vaccinated individuals [3]. Many associations therefore recommend that HTRs be vaccinated before the transplantation, ideally with completion of the vaccine series a minimum of two weeks prior to the transplant [8]. Some heart transplant programs abroad have even mandated immunization before listing for a transplant [13,14] and, subsequently, vaccination rates among HTRs in some countries have been extremely high. The vaccination rate of the Slovenian population against COVID-19 was low in comparison to other European countries, with primary course rates of 57% and 72.6%, respectively. Sociological studies point out that COVID-19 vaccine hesitancy is influenced by several factors, namely economic and political, as well as satisfaction with the health care system and the spread of conspiracy theories [15]. Estimated vaccination rates against COVID-19 in HTRs reported in peer-reviewed literature range from 13.6 to 98.3% [2,16,17]. To the best of our knowledge, we have data neither on the HTR vaccination rate against COVID-19 nor on the clinical course of the disease in HTRs in Slovenia. 

The aim of the present study was for the first time to estimate the epidemiological and clinical characteristics of the course of COVID-19 in HTRs in Slovenia. Our primary objective was to estimate the vaccination rate among HTRs and its influence on clinical presentation, namely length of hospital stay (LOS), rate of acute respiratory insufficiency (ARI), rate of intensive care unit (ICU) admission and rate of in-hospital mortality, respectively. In addition, our secondary objective was to evaluate possible vaccination-hesitant subgroups among the HTRs in Slovenia.

## 2. Materials and Methods

### 2.1. Study Design and Population

**Figure 1:**
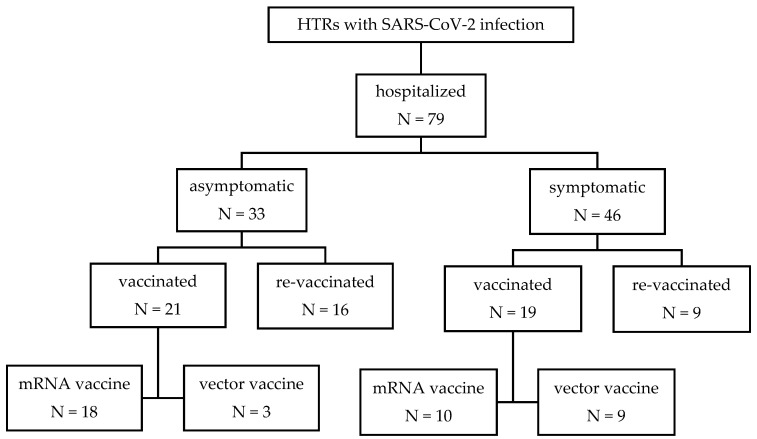
Flow chart of the vaccination and re-vaccination rates among included heart transplant recipients. HTRs—heart transplant recipients, mRNA—Messenger Ribonucleic Acid, N—number of patients.

We conducted a retrospective cohort study including all adult HTRs (≥18 years) who were hospitalized at the Infectious Diseases Department, University Medical Centre Ljubljana, Slovenia, between 1 March 2020 and 31 December 2023 and had a positive nasopharyngeal swab for SARS-CoV-2 (Figure 1). In Slovenia, all heart transplantations are performed at University Medical Centre Ljubljana, where all HTRs are also assessed for any complications (including infections). All patients received standard immunosuppressive therapy, based on the protocol prescribed by the treating cardiologist (corticosteroid, mycophenolate mofetil and calcineurin inhibitor). The diagnosis of SARS-CoV-2 infection was based on a positive polymerase chain reaction (PCR) test from the upper and/or lower respiratory tract, mostly a nasopharyngeal swab. In cases of asymptomatic patients, a cycle threshold (Ct) of less than 30 was considered diagnostic for acute infection, based on the published literature relating to SARS-CoV-2 non-infectivity [18]. According to the national recommendations, which have been changing depending on the current medical knowledge, availability of medications and circulating viral type, patients received different therapies: convalescent plasma, corticosteroids, monoclonal antibodies and remdesivir. Regarding comorbidities, patients were categorized using the Charlson Comorbidity Index (CCI) [19].

### 2.2. Data Retrieval

All vaccination data, including the timing of the vaccination and the type of vaccine used, were obtained from the patient’s electronic medical records. These records also include comprehensive demographic, epidemiological, clinical, laboratory and microbiological data, as well as radiological findings and a full record of the patient’s therapeutic regimen. The study was accessed using following the Declaration of Helsinki and approved by the Slovenian National Medical Ethical Committee on 23 May 2024 (consent number: 0120-171/2024-2711-3).

### 2.3. Definitions

Definition of symptomatic COVID-19 disease—symptomatic COVID-19 was defined as any other hospitalization of patients with clinical signs or symptoms, such as fever, cough, shortness of breath, fatigue or other symptoms of respiratory illness, except hospitalization due to monoclonal antibody, hyperimmune convalescent plasma or remdesivir application in asymptomatic patients.

### 2.4. Statistical Analysis

Statistical significance was assessed using SPSS Statistics 17 (International Business Machines Corporation, Armonk, New York, NY, USA) and a *p* value of 0.05 or lower was considered significant. In the case of a small number of patients in any of the nominal variable categories, the likelihood ratio test was used to test the association between two nominal variables. For nominal variables with more than two categories, the association was tested using the chi-square test or likelihood ratio test in the case of expected frequencies lower than five. The difference between the two groups in a numerical variable was tested with the *t*-test in case the variable was normally distributed across groups; otherwise, the Mann–Whitney U test was used. Bivariate Kendall’s tau correlation was used to study the relationship between different factors. We used the mean and standard deviation as measures of descriptive statistics, as all continuous variables were normally distributed.

## 3. Results

The study included 79 HTRs with a mean age of 62.5 years (±9.8), 72.2% (57) of patients being male. The mean CCI was 3.3 (±1.8), and the mean time from heart transplantation was 5.66 years (±5.29). The average time from vaccination to hospital admission was 402 days (±232), with an average (LOS) of 10.4 days (±11.9). A total of 58.2% (46) of patients had symptomatic COVID-19 at the time of hospital admission. ARI was present in 35.4% of patients, and 7.6% were admitted to ICU. The overall in-hospital mortality rate was 5.1%, with an ICU mortality rate of 66%. At the time of hospital admission, 64.5% (51) of patients were receiving corticosteroid therapy. Regarding COVID-19 treatment, 16.5% (13) of patients received hyperimmune convalescent plasma, 24.1% (19) were treated with monoclonal antibodies (mAb), 78.4% (62) received remdesivir and 24.0% (19) corticosteroids. Regarding vaccination, 63.3% (50) of patients had received at least one dose of COVID-19 vaccine. Among the vaccinated patients, 70.0% (28) had received an mRNA-based vaccine and 30.0% (12) had received a vector-based vaccine. Additionally, 43.1% (34) of patients received a booster dose of the vaccine, with 31.6% (25) having received the booster before hospitalization. The demographic, clinical and vaccination characteristics of included patients are presented in Table 1.

### 3.1. Comparison Between Vaccinated and Unvaccinated Patients

The mean age of vaccinated patients was 65.6 years, significantly older than the unvaccinated group, which had a mean age of 59.3 years (*p* = 0.004). The CCI was higher in the vaccinated group (3.7 ± 1.8) compared to the unvaccinated group (2.8 ± 1.7), with a statistically significant difference (*p* = 0.018). There was no significant difference in the time from heart transplantation, the LOS, ARI, the ICU admission rate and the in-hospital mortality rate. Treatment with hyperimmune convalescent plasma was significantly more commonly used in the unvaccinated group (30.8%, twelve patients) compared to the vaccinated group (2.5%, one patient) (*p* = 0.001). There were no significant differences in the use of mAb, remdesivir or corticosteroids. We performed a subgroup analysis of the vaccinated patients based on the type of vaccine they received. A total of 28 patients received an mRNA-based vaccine and 12 received a vector-based vaccine. There were no significant differences between the groups in terms of age (*p* = 0.371), gender (*p* = 0.804) or CCI (*p* = 0.327). The time from heart transplantation was longer in the mRNA group (7.3 years ± 4.6) compared to the vector group (4.4 years ± 5.0), but this difference did not reach statistical significance (*p* = 0.085). The time from vaccination to hospital admission was similar between the two groups (388 days ± 215 for mRNA vs. 431 days ± 218 for vector, *p* = 0.603). LOS was significantly shorter in the mRNA group (6.6 days ± 9.3) compared to the vector group (14.3 days ± 15.3) (*p* = 0.011). The incidence of ARI was lower in the mRNA group (21.4%, six patients) compared to the vector group (41.7%, five patients), but this difference was not statistically significant (*p* = 0.189). There were no significant differences in ICU admissions (7.1% for mRNA vs. 16.7% for vector, *p* = 0.358) or in-hospital mortality (7.1% for mRNA vs. 8.3% for vector, *p* = 0.896). Treatment patterns did not differ significantly between groups, including the use of hyperimmune convalescent plasma, monoclonal antibodies, remdesivir and corticosteroids.

A subgroup analysis was conducted for patients with symptomatic COVID-19 disease, which included 27 patients. The mean age of vaccinated patients was significantly higher (66.7 years ± 7.2) compared to unvaccinated patients (61.2 years ± 7.7) (*p* = 0.017). There were no significant differences between the groups regarding gender, CCI or time from heart transplantation. Among these patients, ARI was observed in 57.9% (11) of vaccinated patients compared to 63.0% (17) of unvaccinated patients (*p* = 0.729). The ICU admission rate was 21.1% (4) for vaccinated patients and 7.4% (2) for unvaccinated patients (*p* = 0.176). The in-hospital mortality rate was higher in vaccinated patients (15.8%) compared to unvaccinated patients (3.7%), though this difference was not statistically significant (*p* = 0.152). The use of hyperimmune convalescent plasma was significantly higher in the unvaccinated group (44.4%, twelve patients) compared to the vaccinated group (5.3%, one patient) (*p* = 0.004). There were no significant differences in the use of mAb, remdesivir or corticosteroids.

Lastly, a subgroup analysis was performed, comparing patients who received a booster dose of the vaccine with those who did not. The mean age was similar between groups, but the gender distribution differed significantly, with 92.0% (23) of patients receiving the booster being male, compared to 53.3% (8) in the no-booster group (*p* = 0.005). There was a trend toward longer LOS in the no-booster group (12.2 days ± 12.5) compared to the booster group (7.0 days ± 11.1), and this difference was statistically significant (*p* = 0.030). Other clinical parameters, including respiratory insufficiency (24.0% with booster vs. 33.3% without, *p* = 0.522), ICU admission (12.0% with booster vs. 6.7% without, *p* = 0.586) and in-hospital mortality (12.0% with booster vs. 0% without, *p* = 0.163), did not show significant differences. Treatment regimens were similar between groups, with no significant differences in the use of hyperimmune convalescent plasma, monoclonal antibodies, remdesivir and corticosteroids.

Comparison between vaccinated HTRs based on gender revealed no statistically significant differences (*p* = 0.283), while comparison between re-vaccinated HTRs based on gender revealed that males were re-vaccinated statistically significantly more often (*p* = 0.007). 

### 3.2. Descriptive Analysis of Non-Survivors

Four out of 79 patients died (5%); all were hospitalized due to severe COVID-19 pneumonia, three were vaccinated and one was not. Their mean age was 71.3 years, the average sum of CCI was 4.5 and the average time from heart transplantation was 10 years. Two patients died because of COVID-19-related complications (severe pneumonia with pulmonary fibrosis; COVID-19-associated pulmonary aspergillosis with diffuse bleeding) and two patients due to non-COVID-19-related complications (septic shock with multiorgan failure; hemorrhagic shock due to retroperitoneal bleeding).

### 3.3. Analysis of Heart Transplant Recipients Based on the Time from Vaccination to Hospital Admission

We performed a comparison between HTRs in terms of outcome based on the time from vaccination to hospital admission. The results are presented in Table 2. Only HTRs who were vaccinated before the hospitalization were included in the analysis. Patients were grouped based on the time from vaccination to hospital admission: <3 months, 3–6 months, 6–12 months and >12 months. There were no significant differences among groups in terms of clinical outcomes. The overall *p*-value for LOS was 0.551, for ARI 0.509, for ICU admission 0.426 and for in-hospital mortality 0.613, respectively. Regarding vaccine type, the majority of patients in all groups received the mRNA-based vaccine—75% of those in the <3 months group, 40% in the 3–6 months group, 88.9% in the 6–12 months group and 66.7% in the >12 months group, respectively. The *p*-value of 0.288 suggests no significant difference in vaccination type distribution between groups.

### 3.4. Analysis of Heart Transplant Recipients Based on the Time from Their Heart Transplant

**Figure 2:**
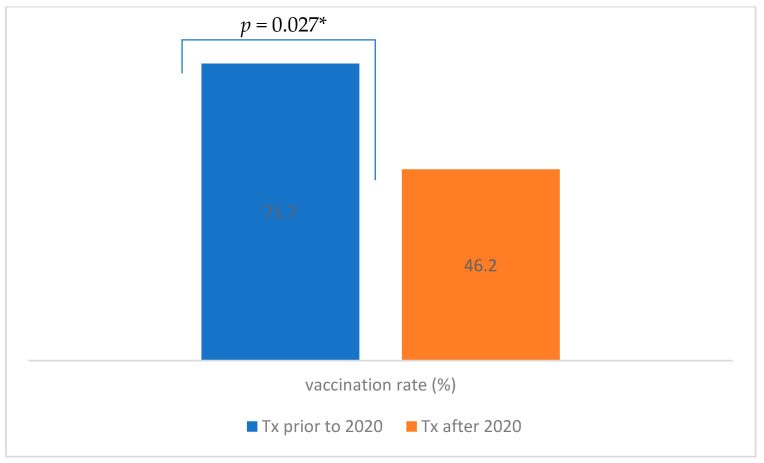
Comparison between vaccination rates of heart transplant recipients that received their transplant before the start or during the pandemic. Tx prior to 2020 = heart transplant before year 2020; tx after 2020 = heart transplant in and after year 2020. * Significant at *p* < 0.05.

We performed a comparison between HTRs in terms of outcome based on the time from their heart transplant. The results are presented in Table 3. Only HTRs who were vaccinated before the hospitalization were included in the analysis. The comparison between HTRs vaccinated before or during the pandemic is represented in Figure 2. Patients were divided into two groups: those who had a heart transplant less than one year before hospital admission (N = 23) and those who had a transplant more than one year prior to admission (N = 56). There were no significant differences across groups in terms of clinical outcomes, and the overall result for different outcomes were as follows: LOS 11.6 days vs. 10.0 days (*p* = 0.918), ARI 26.1% vs. 39.3% (*p* = 0.265), ICU admissions 8.7% vs. 7.1% (*p* = 0.813) and in-hospital mortality 0 vs. 7.1% (*p* = 0.188).

### 3.5. Comparison of Patients with Symptomatic and Asymptomatic COVID-19 Disease

We performed a comparison between symptomatic and asymptomatic HTRs. The results are presented in Table 4. We compared the HTRs with symptomatic (N = 46) and asymptomatic (N = 33) clinical courses. The mean age (63.5 vs. 61.2 years, *p* = 0.303), gender distribution (male gender 67.4% vs. 78.8%, *p* = 0.265), CCI (3.5 vs. 3.0, *p* = 0.141) and time from heart transplantation (5.2 vs. 6.4 years, *p* = 0.538) were all similar between groups. A higher proportion of asymptomatic patients were vaccinated compared to symptomatic patients—63.6% (21 patients) vs. 41.3% (19 patients); this difference was statistically significant (*p* = 0.050). A significantly higher proportion of asymptomatic patients received the mRNA-based vaccine (85.7% vs. 52.6%, *p* = 0.023), as well as a booster dose (48.5% vs. 19.6%, *p* = 0.006). In terms of clinical outcomes, symptomatic patients had a significantly higher rate of ARI (60.9%; *p* < 0.001) and a significantly higher proportion were admitted to the ICU (13%; *p* = 0.031). The in-hospital mortality rate was also higher in symptomatic patients (8.7%) compared to asymptomatic patients (0%), although this difference was not statistically significant (*p* = 0.082). When comparing treatment regimens between the two groups, we observed that 28.3% of symptomatic patients were treated with hyperimmune convalescent plasma, while none of the asymptomatic patients received this treatment. This difference was statistically significant (*p* = 0.001). A total of 41.3% of symptomatic patients received corticosteroids, while none of the asymptomatic patients were treated with corticosteroids. This difference was also statistically significant (*p* = 0.000). We found no significant differences in the use of mAb and remdesivir.

### 3.6. Bivariate Correlation Analysis

We performed a bivariate correlation analysis for all measures. The results are presented in Table 5. The correlation analysis revealed several significant relationships between patient characteristics, treatments and outcomes. Age and CCI showed a significant positive correlation (r = 0.50, *p* < 0.01), indicating that older patients tend to have more comorbidities. Additionally, CCI was positively associated with LOS (r = 0.18, *p* < 0.05), suggesting that patients with more comorbidities tend to stay longer in the hospital. Symptomatic COVID-19 disease (SYMP) was strongly correlated with LOS (r = 0.73, *p* < 0.01), meaning symptomatic patients generally had longer hospital stays. Moreover, SYMP was associated with ARI (r = 0.63, *p* < 0.01) and steroid use (r = 0.48, *p* < 0.01), reflecting that symptomatic patients were more likely to experience respiratory complications and receive steroid treatment. The relationship between ICU admission and in-hospital mortality (MORT) was also significant (r = 0.81, *p* < 0.01), demonstrating that patients admitted to the ICU had a higher likelihood of dying during hospitalization. 

Vaccination was negatively correlated with symptomatic COVID-19 disease (r =−0.19, *p* < 0.05), indicating that vaccinated patients were less likely to develop symptomatic disease. mAb therapy was negatively correlated with vaccination status (r = −0.25, *p* < 0.05), meaning vaccinated patients were less likely to receive mAb. Similarly, the mRNA vaccine was negatively associated with LOS (r = −0.35, *p* < 0.05), suggesting that vaccinated patients who received mRNA vaccines tended to have a shorter LOS. Lastly, time since vaccination (T-VAC) showed a negative correlation with the use of mAb (r = −0.33, *p* < 0.05), implying that patients who had been vaccinated longer ago were more likely to receive mAb.

## 4. Discussion

HTRs require lifelong immunosuppression, representing an increased risk of COVID-19 infection, as well as a much higher risk of consequent hospitalization and mortality. Data show a significantly more favorable course in vaccinated individuals [1,2,3,4]. Also in our cohort, an asymptomatic course was more often observed in vaccinated HTRs, as several other studies have similarly confirmed in immunocompromised and non-immunocompromised vaccine recipients. In general, vaccinated patients tend to have less severe COVID-19-related respiratory failure, a better clinical course, a lower mortality rate and a higher hospital discharge rate [3,20]. Furthermore, an asymptomatic clinical course was more often observed in those vaccinated with an mRNA-based vaccine and those receiving a vaccine booster dose. These findings are in accordance with already published data as well; 135 persons vaccinated with an mRNA-based vaccine had a stronger immune response to the vaccination than the 33 recipients of a vector-based vaccine [21]. A retrospective cohort of data from the United States National COVID Cohort Collaborative, including more than two million participants, of whom 17.5 % were immunocompromised, confirm that booster mRNA-based vaccine doses remain effective against severe COVID-19, regardless of patients’ immune status [22]. We also demonstrated that COVID-19 booster vaccination was associated with a significantly shorter LOS, despite re-vaccinated HTRs having a higher CCI, which approached statistical significance. Our findings align with prospective research underscoring the importance of booster vaccination in HTRs to reduce severe outcomes and hospitalization duration following COVID-19 infection [10]. 

In our cohort, unvaccinated HTRs were generally younger and had a lower CCI than those vaccinated against COVID-19. However, unvaccinated HTRs were more frequently treated with COVID-19 hyperimmune convalescent plasma. Vaccinated HTRs had at least partial immunity, while unvaccinated patients lacked protection against SARS-CoV-2 infection. In cases where an HTR was unvaccinated, we were more likely to choose treatment with hyperimmune convalescent plasma, due to concerns about a potentially more severe course of COVID-19. The use of hyperimmune convalescent plasma provides immediate passive immunity via neutralizing antibodies against SARS-CoV-2. When administered early in the disease course (within 72 hours of symptom onset), it can offer prompt protection, potentially reducing the risk of disease progression, hospitalization and mortality [23].

Not only did HTRs vaccinated with a vector-based vaccine have a longer LOS compared to those vaccinated with an mRNA-based vaccine, but they also required ICU admission more frequently. Although the age difference between the two groups was not statistically significant, those HTRs receiving vector-based vaccines had higher CCI scores, indicating a greater burden of comorbidities that may have influenced LOS and ICU admission. Although the differences were not statistically significant, the shorter time from heart transplantation to hospital admission, along with the longer interval from vaccination to hospital admission in patients vaccinated with vector-based vaccines, suggests a trend. This may indicate that patients receiving vector-based vaccines were more likely vaccinated during a period of more intense immunosuppression compared to those vaccinated with mRNA vaccines. Memenga et al. found in a prospective study including 91 participants that advanced patient age and shorter time since heart transplantation were associated with lower concentrations of anti-SARS-CoV-2 spike IgG antibodies after three vaccine doses, but did not observe differences in antibody levels between the vaccine types (mRNA versus vector-based vaccine) [10]. Conversely, Huang et al. reported in a Taiwanese prospective cohort of 824 subjects that higher CCI values correlated with lower levels of IgG antibodies against the SARS-CoV-2 spike protein, which may also explain the prolonged hospitalization in patients vaccinated with vector vaccines due to greater comorbidity and, consequently, a poorer vaccine response [24].

There was a statistically significant higher re-vaccination rate in male HTRs, probably reflecting the previously described phenomenon that, compared to men, women had less trust in vaccines [25,26,27]. The average time from vaccination to hospital admission in our cohort was more than a year, which confirms the sense of regular re-vaccinations for immunocompromised patients, according to the foreign and Slovenian recommendations [28]. 

Immunocompromised patients remain at an elevated risk of death from COVID-19, especially those who are critically ill [29], and the same applies to those who need treatment in the ICU [1,4,30]. Retrospective data from Netherlands, including 54 HTRs, suggest that HTRs were at increased risk of complicated COVID-19, and all-cause mortality was higher than in our cohort (14% vs. 5.1%), but their results were from the pre-vaccination era [31], which may suggest that mortality among our patients was lower due to the vaccination effect. Similarly, Peters et al. showed in a single-center case-control study including 436 HTRs the association of COVID-19 vaccination with risk of infection, hospitalization and death among HTRs in a US heart transplant program, and the rates were significantly lower among those vaccinated [3]. In terms of the presence of immunocompromising conditions and vaccination status, Stupica et al. found that patients in Slovenia admitted to hospital during the Omicron period had similar odds of progressing to critically severe disease to those admitted during the Delta period. Patients completing at least primary vaccination had lower odds of progression to critically severe disease and had a shorter LOS than unvaccinated patients [32]. On the other hand, in our ICU the cohort mortality rate, despite high vaccination status, was higher in comparison to other reports; meta-analyses of patients requiring ICU admission and mechanical ventilation reached mortality rates up to 50% [33]. In our opinion, the reason could be that our ICU patients were older and had several comorbidities. Additionally, the cohort of our patients treated in the ICU was very small. Older persons, those with a higher CCI and the immunocompromised were statistically significantly more often vaccinated. 

Vaccine hesitancy has been ranked by the World Health Organization among the top 10 threats to global health [34]. The results of our study suggest that the vaccination rate among HTRs in our tertiary center was lower than reported from abroad and from the general Slovenian population. Some foreign reports also suggest a low acceptance rate of COVID-19 vaccines in HTRs [16,35], while, on the other hand, reports from Argentina show extremely high rates of vaccination among HTRs, which resulted in no confirmed COVID-19 cases during the Delta variant circulation [2]. There is a paucity of data regarding vaccination hesitancy factors among HTRs in Slovenia. Our analysis suggests that younger HTRs and those receiving transplants after the pandemic are more hesitant to get vaccinated, and female HTRs accept a booster dose less frequently. We assume that the hesitancy factors in our cohort are similar to those in the general Slovenian population. A cross-sectional survey from Slovenia regarding COVID-19 vaccination intention, confidence and hesitancy among the working population revealed a 58% vaccination rate of the respondents, while 11.3% refused to be vaccinated. The hesitant group was most often deterred by distrust of COVID-19 vaccines, including the inability to choose a vaccine and the fear of side effects [36]. Reports suggest that mRNA vaccines were related to the adverse effect of myocarditis [37], and although studies have shown a significantly higher chance of myocarditis after SARS-CoV-2 infection than vaccination, some people were deterred from COVID-19 vaccination. There are no data on vaccination-related myocarditis in HTRs available in peer-reviewed literature. Cardiac inflammation has been suggested to be regulated by the sympathetic nervous system. A mice model on myocardial damage in myocardial infarction (MI) showed that cardiac sympathetic denervation significantly attenuated chronic consequences of MI, including myocardial inflammation, myocyte hypertrophy and overall cardiac dysfunction. Further studies on animal models should be performed, but heart denervation might be the reason for there being no reports on myocarditis in HTRs [38]. An online survey by the National Institute of Public Health (NIPH) from Slovenia, dated December 2020, revealed the attitudes of the population regarding COVID-19 vaccination and the factors that affected these attitudes. A greater intention to get vaccinated was associated with men and older respondents, as was found in our cohort as well. Others more likely to get vaccinated were physicians and medical students, respondents who had got the influenza vaccination, those who knew someone who had been hospitalized or had died from COVID-19, and those who had more trust in experts, institutions and vaccines [39]. According to the NIPH publication, more than 71% of the respondents were vaccinated against COVID-19 in the year 2022, which is in concordance with data from the Slovenian Electronic Registry of Vaccinated Persons (eRCO). The main reasons to get vaccinated were to prevent a more severe course, to protect the health of relatives and to protect one’s own health [40]. Data from a US transplant center suggest that 82 (96.5%) HTRs received a COVID-19 vaccine, with the primary motivating factor being to protect their own health [17]. In our study, we found that the HTR vaccination rate was significantly higher in those receiving a transplant before the year 2020. The reason might be due to global vaccine hesitancy with a surge in misinformation and conspiracy theories against vaccination observed during the COVID-19 pandemic. On the other hand, immunocompromised patients were among the first to be vaccinated against COVID-19 in our country, and soon after, reports regarding the side effect of myocarditis appeared. 

Our study has several limitations. The study was conducted retrospectively and included a relatively small cohort of patients, which reduces the statistical power of our findings. Due to the relatively small sample size, there is the consideration that methodological recommendations for retrospective study sampling present a potential although minimal possibility that the detection of significant differences could be impacted [41]. Regarding the clinical course, we did not assess antibody to SARS-CoV-2 nucleocapsid protein levels on admission and thus cannot entirely exclude the possibility that some of the patients included in the study had previously contracted SARS-CoV-2, which could have also influenced the clinical course of the COVID-19 episode for which they were hospitalized during the study period, as the research is retrospective. Reinfection with SARS-CoV-2 in SOT patients is often characterized by a milder clinical course compared to primary infections, with reduced incidence of severe complications such as ARI [42]. Antibodies against the nucleocapsid protein, which are generated following recovery from COVID-19, tend to appear in lower concentrations and decline more rapidly over time compared to the general population. The antibody levels are also influenced by the severity of the COVID-19 course. Consequently, SOT recipients who experienced a mild infection could have a negative antibody test result for the nucleocapsid protein [43]. An added value would be if our HTRs had completed a questionnaire on the reasons for and against vaccination and their social status. However, the advantage of our research being conducted at a single center is the comprehensive data capture and the uniform treatment regimen for HTRs with COVID-19. The research was conducted in a single center, but it should be emphasized that heart transplants in Slovenia are performed only at UMC Ljubljana, where HTRs come for regular check-ups, as well. As a rule, all Slovenian HTRs who contracted COVID-19 were treated in our institution, so we assume that all HTRs from Slovenia with SARS-CoV-2 infection who required hospital treatment during the study period were included in the study.

## 5. Conclusions

This is the first study presenting the epidemiological and clinical characteristics of the course of COVID-19 in HTRs in Slovenia, as well as estimating the vaccination rate at 63.3%. Our results suggest that vaccination is crucial to preventing more severe clinical presentations of COVID-19 in HTRs in Slovenia. In vaccinated HTRs, the clinical course of COVID-19 was more often asymptomatic, with lower incidence of ARI and need for ICU treatment, respectively; furthermore, in recipients of mRNA-based vaccines and those re-vaccinated, the LOS was shorter. For the first time, we evaluated vaccination-hesitant subgroups among the HTRs in Slovenia. Our results suggest that among HTRs in Slovenia, younger patients and those receiving a transplant after the pandemic, as well as women, are more hesitant to receive a COVID-19 vaccination, representing a subgroup which should be addressed more actively regarding vaccination.

The spread of expert advice and the creation of a safe and respectful communication environment to promote vaccination and debunk vaccination myths is crucial in health and vaccination promotion, especially among the immunocompromised, who are more prone to a severe course of COVID-19. Studies have shown that stimulation of positive feelings plays a significant role in overcoming vaccine hesitancy. HTRs are more susceptible to COVID-19, especially a severe course, so it is crucial to promote high vaccination rates, especially in those who are more hesitant. 

## Figures and Tables

**Table 1:** Demographic, clinical and vaccination characteristics of included patients and comparison between vaccinated and unvaccinated heart transplant recipients.

Title		N = 79	
Mean age (years) (±SD)		62.5 (±9.8)	
Male gender (%)		57 (72.2)	
CCI (±SD)		3.3 (±1.8)	
Time from heart transplantation (years) (±SD)		5.66 (±5.29)	
Time from vaccination to hospital admission (days) (±SD)		402 (±232)	
Length of hospital stay in days (±SD)		10.4 (±11.9)	
No. of patients with symptomatic COVID-19 disease (%)		46 (58.2)	
No. of patients with respiratory insufficiency (%)		28 (35.4)	
No. of patients admitted to ICU (%)		6 (7.6)	
In-hospital mortality rate (%)		4 (5.1)	
ICU mortality rate (%)		4 (66.7)	
No. of patients on corticosteroid therapy at hospital admission (%)		51 (64.5)	
No. of patients treated with			
- hyperimmune convalescent plasma (%)		13 (16.5)	
- mAb (%)		19 (24.1)	
- remdesivir (%)		62 (78.4)	
- corticosteroid (%)		19 (24.0)	
No. of patients vaccinated (%)		50 (63.3)	
No of patients vaccinated before hospitalization (%)		40 (50.6)	
No. of patients receiving mRNA-based vaccine (%)		28 (70.0)	
No. of patients receiving vector-based vaccine (%)		12 (30.0)	
No. of patients receiving a booster dose of vaccine (%)		34 (43.1)	
No. of patients receiving a booster dose of vaccine before hospitalization (%)		25 (31.6)	
Comparison of Vaccinated and Unvaccinated Heart Transplant Recipients			
	**Vaccinated** **N = 40**	**Unvaccinated** **N = 39**	
Mean age (years) (±SD)	65.6 (±8.9)	59.3 (±9.8)	*p* = 0.004 *
Male gender (%)	31 (77.5)	26 (66.7)	*p* = 0.283
CCI (±SD)	3.7 (±1.8)	2.8 (±1.7)	*p* = 0.018 *
Time from heart transplantation (years) (±SD)	6.4 (±4.8)	4.9 (±5.7)	*p* = 0.194
Length of hospital stay (days) (±SD)	9.0 (±11.8)	12.0 (±12.0)	*p* = 0.106
No. of patients with respiratory insufficiency (%)	11 (27.5)	17 (43.6)	*p* = 0.135
No. of patients admitted to ICU (%)	4 (10.0)	2 (5.1)	*p* = 0.414
In-hospital mortality rate (%)	3 (7.5)	1 (2.6)	*p* = 0.317
No. of patients (%) treated with			
- hyperimmune convalescent plasma	1 (2.5)	12 (30.8)	*p* = 0.001 *
- mAb	9 (22.5)	10 (25.6)	*p* = 0.744
- remdesivir	34 (85.0)	28 (71.8)	*p* = 0.153
- corticosteroid	9 (22.5)	10 (25.6)	*p* = 0.744
Subgroup analysis of heart transplant recipients receiving an mRNA-based vaccine or a vector-based vaccine			
	**mRNA vaccine** **N = 28**	**Vector vaccine** **N = 12**	
Mean age (years) (±SD)	66.5 (±9.8)	63.7 (±6.4)	*p* = 0.371
Male gender (%)	22 (78.6)	9 (75.0)	*p* = 0.804
CCI (±SD)	3.5 (±2.0)	4.2 (±1.3)	*p* = 0.327
Time from heart transplantation (years) (±SD)	7.3 (±4.6)	4.4 (±5.0)	*p* = 0.085
Time from vaccination to hospital admission (days) (±SD)	388 (±215)	431 (±218)	*p* = 0.603
Length of hospital stay (days) (±SD)	6.6(±9.3)	14.3 (±15.3)	*p* = 0.011 *
No. of patients with respiratory insufficiency (%)	6 (21.4)	5 (41.7)	*p* = 0.189
No. of patients admitted to ICU (%)	2 (7.1)	2 (16.7)	*p* = 0.358
In-hospital mortality rate (%)	2 (7.1)	1 (8.3)	*p* = 0.896
No. of patients (%) treated with			
- hyperimmune convalescent plasma	1 (3.6)	0	*p* = 0.507
- mAb	6 (21.4)	3 (25.0)	*p* = 0.804
- remdesivir	24 (85.7)	10 (83.3)	*p* = 0.847
- corticosteroid	5 (17.9)	4 (33.3)	*p* = 0.283
Subgroup analysis of vaccinated and unvaccinated heart transplant recipients with symptomatic COVID-19			
	**Vaccinated** **N = 19**	**Unvaccinated** **N = 27**	
Mean age (years) (±SD)	66.7 (±7.2)	61.2 (±7.7)	*p* = 0.017 *
Male gender (%)	14 (73.7)	17 (63.0)	*p* = 0.445
CCI (±SD)	3.8 (±1.4)	3.2 (±1.6)	*p* = 0.154
Time from heart transplantation (years) (±SD)	5.9 (±4.7)	4.6 (±4.7)	*p* = 0.370
No. of patients with respiratory insufficiency (%)	11 (57.9)	17 (63.0)	*p* = 0.729
No. of patients admitted to ICU (%)	4 (21.1)	2 (7.4)	*p* = 0.176
In-hospital mortality rate, %	3 (15.8)	1 (3.7)	*p* = 0.152
No. of patients (%) treated with			
- hyperimmune convalescent plasma	1 (5.3)	12 (44.4)	*p* = 0.004 *
- mAb	7 (36.8)	6 (22.2)	*p* = 0.278
- remdesivir	15 (78.9)	18 (66.7)	*p* = 0.362
- corticosteroid	9 (47.4)	10 (37.0)	*p* = 0.483
Subgroup analysis of heart transplant recipients who received a booster dose of vaccine and those who did not			
	**Booster** **N = 25**	**No booster** **N = 15**	
Mean age (years) (±SD)	66.3 (±10.3)	64.5 (±6.3)	*p* = 0.556
Male gender (%)	23 (92.0)	8 (53.3)	*p* = 0.005 *
CCI (±SD)	4.2 (±1.9)	3.0 (±1.5)	*p* = 0.052
Time from heart transplantation (years) (±SD)	7.0 (±5.2)	4.2 (±5.5)	*p* = 0.372
Length of hospital stay (days) (±SD)	7.0 (±11.1)	12.2 (±12.5)	*p* = 0.030 *
No. of patients with respiratory insufficiency (%)	6 (24.0)	5 (33.3)	*p* = 0.522
No. of patients admitted to ICU (%)	3 (12.0)	1 (6.7)	*p* = 0.586
In-hospital mortality rate, %	3 (12.0)	0	*p* = 0.163
No. of patients (%) treated with			
- hyperimmune convalescent plasma	1 (4.0)	0	*p* = 0.433
- mAb	4 (16.0)	5 (33.3)	*p* = 0.204
- remdesivir	22 (88.0)	12 (80.0)	*p* = 0.493
- corticosteroid	5 (20.0)	4 (26.7)	*p* = 0.625

CCI—Charlson Comorbidity Index; ICU—intensive care unit; mAb—monoclonal antibodies; mRNA—Messenger Ribonucleic Acid; N—number of patients; SD—standard deviation. * Significant at *p* < 0.05.

**Table 2:** Comparison between heart transplant recipients based on time from vaccination to hospital admission.

	<3 Months N = 4	3–6 Months N = 5	6–12 Months N = 9	>12 Months N = 21	
Length of hospital stay (days) (±SD)	4.5 (±1.3)	11.8 (±7.8)	11.6 (±16.0)	7.4 (±11.6)	*p* = 0.551
No. of patients with respiratory insufficiency (%)	2 (50.0)	2 (40.0)	2 (22.2)	4 (19.0)	*p* = 0.509
No. of patients admitted to ICU (%)	0	0	0	3 (14.3)	*p* = 0.426
In-hospital mortality rate (%)	0	0	0	9.5	*p* = 0.613
No. of patients (%) vaccinated with					
- mRNA-based vaccine	3 (75.0)	2 (40.0)	8 (88.9)	14 (66.7)	*p* = 0.288
- vector-based vaccine	1 (25.0)	3 (60.0)	1 (11.1)	7 (33.3)	

ICU—intensive care unit; mRNA—Messenger Ribonucleic Acid; N—number of patients; SD—standard deviation.

**Table 3:** Comparison between heart transplant recipients with a recent (less than one year prior to admission) heart transplant and the rest of cohort.

	Transplant Less Than One Year Before Admission N = 23	Transplant More Than One Year Before Admission N = 56	
Length of hospital stay in days (±SD)	11.6 (±14.3)	10.0 (±10.9)	*p* = 0.918
No. of patients with respiratory insufficiency (%)	6 (26.1)	22 (39.3)	*p* = 0.265
No. of patients admitted to ICU (%)	2 (8.7)	4 (7.1)	*p* = 0.813
In-hospital mortality rate (%)	0	4 (7.1)	*p* = 0.188

ICU—intensive care unit; N—number of patients; SD—standard deviation.

**Table 4:** Comparison between heart transplant recipients with symptomatic and asymptomatic COVID-19.

	Symptomatic N = 46	Asymptomatic N = 33	
Mean age (years) (±SD)	63.5 (±7.9)	61.2 (±12.0)	*p* = 0.303
Male gender (%)	31 (67.4)	26 (78.8)	*p* = 0.265
CCI (±SD)	3.5 (±1.5)	3.0 (±2.1)	*p* = 0.141
Time from heart transplantation (years) (±SD)	5.2 (±4.7)	6.4 (±6.1)	*p* = 0.538
No. of patients vaccinated (%)	19 (41.3)	21 (63.6)	*p* = 0.050 *
No. of patients receiving a booster dose of vaccine (%)	9 (19.6)	16 (48.5)	*p* = 0.006 *
No. of patients receiving an mRNA-based vaccine (%)	10 (52.6)	18 (85.7)	*p* = 0.023 *
No. of patients receiving a vector-based vaccine (%)	9 (47.4)	3 (14.3)	
No. of patients with respiratory insufficiency (%)	28 (60.9)	0	*p* = 0.000 *
No. of patients admitted to ICU (%)	6 (13.0)	0	*p* = 0.031 *
In-hospital mortality rate (%)	4 (8.7)	0	*p* = 0.082
No. of patients (%) treated with			
- hyperimmune convalescent plasma	13 (28.3)	0	*p* = 0.001 *
- mAb	13 (28.3)	6 (18.2)	*p* = 0.301
- remdesivir	33 (71.7)	29 (87.9)	*p* = 0.085
- corticosteroid	19 (41.3)	0	*p* = 0.000 *

CCI—Charlson Comorbidity Index; ICU—intensive care unit; mAb—monoclonal antibodies; mRNA—Messenger Ribonucleic Acid; N—number of patients, SD—standard deviation. * Significant at *p* < 0.05.

**Table 5:** Bivariate correlation for all measures (Kendall’s tau correlation coefficient).

Measure	1.	2.	3.	4.	5.	6.	7.	8.	9.	10.	11.	12.	13.	14.	15.	16.	17.
1. Age	1.00																
2. Gender	0.14	1.00															
3. CCI	0.50 **	0.23 *	1.00														
4. T-TX	0.33 **	0.17	0.19 *	1.00													
5. T-VAC	−0.10	−0.13	−0.15	−0.07	1.00												
6. LOS	0.09	−0.14	0.18 *	0.02	−0.11	1.00											
7. SYMP	0.04	−0.13	0.15	−0.06	−0.19	0.73 **	1.00										
8. ARI	0.12	−0.07	0.07	0.07	−0.20	0.59 **	0.63 **	1.00									
9. ICU	0.07	−0.04	0.09	0.05	0.17	0.32 **	0.24 *	0.39 **	1.00								
10. MORT	0.17	0.02	0.16	0.17	0.05	0.25 **	0.20	0.31 **	0.81 **	1.00							
11.HCP	−0.08	0.05	0.07	−0.02	−0.22	0.35 **	0.38 **	0.24 *	−0.13	−0.10	1.00						
12. mAb	−0.05	0.09	−0.02	−0.05	−0.33 *	0.01	0.12	0.08	−0.05	0.01	−0.25 *	1.00					
13. remde	0.06	0.02	0.02	−0.06	0.29 *	−0.11	−0.19	0.00	0.03	−0.02	0.15	−0.43	1.00				
14. stero	0.02	0.14	0.10	0.16	0.33 *	0.44 **	0.48 **	0.76 **	0.29 *	0.41 **	0.15	0.10	−0.14	1.00			
15. VAC.	0.28 **	0.12	0.25 *	0.18	A	−0.16	−0.22	−0.17	0.09	0.11	−0.38 **	−0.04	0.16	−0.04	1.00		
16. mRNA	0.19	0.04	−0.21	0.26	−0.09	−0.35 *	−0.36 *	−0.21	−0.15	−0.02	0.11	−0.04	0.03	−0.17	a	1.00	
17.R-VAC	0.25 **	0.30 **	0.31 **	0.18	0.05	−0.25 **	−0.31 **	−0.16	0.11	0.22	−0.23 *	−0.13	0.16	−0.06	0.67 **	0.06	1.00

* Significant at *p* < 0.05; ** significant at *p* < 0.01. Abbreviations: CCI—Charlson Comorbidity Index; T-TX—time from heart transplantation; T-VAC—time from vaccination to hospital admission; LOS—length of hospital stay; SYMP—symptomatic COVID-19 disease; ARI—acute respiratory insufficiency; ICU—intensive care unit admission; MORT—in-hospital mortality; HCP—treatment with hyperimmune convalescent plasma; mAb—monoclonal antibodies application; remde—treatment with remdesivir; stero—treatment with steroids; VAC—vaccinated; mRNA—vaccinated with mRNA vaccine; R-VAC—re-vaccinated. a—cannot be computed, as part of the same measure.

## Data Availability

Dataset available on request from the authors.
